# Sulfide Species Optical Monitoring by a Miniaturized Silicon Photomultiplier

**DOI:** 10.3390/s18030727

**Published:** 2018-02-28

**Authors:** Salvatore Petralia, Emanuele Luigi Sciuto, Maria Francesca Santangelo, Sebania Libertino, Maria Anna Messina, Sabrina Conoci

**Affiliations:** 1 STMicroelectronics, Stradale Primosole 50, 95121 Catania, Italy; 2 Diploma of Physics and Astronomy, University of Catania, 95100 Catania, Italy; elsciuto@unict.it; 3 CNR-IMM Sede, Strada VIII Z.I. 5, 95121 Catania, Italy; Francesca.Santangelo@imm.cnr.it (M.F.S.); Sebania.Libertino@imm.cnr.it (S.L.); 4 Azienda Policlinico Universitario, Via S. Sofia 78, 95100 Catania, Italy; mmessina@unict.it; 5 Centro Speleologico Etneo, Via Valdisavoia 5, 95123 Catania, Italy

**Keywords:** sulfide, silicon photomultiplier, water

## Abstract

The monitoring of water-soluble pollutants is receiving a growing interest from the scientific community. In this context, sulfide anion species S^2−^ and HS^−^ are particularly relevant since they can cause acute and chronic toxicity including neurological effects and at high concentrations, even death. In this study, a new strategy for fast and sensitive optical detection of sulfide species in water samples is described. The method uses an integrated silicon photomultiplier (SiPM) device coupled with the appropriate analytical strategy applied in a plastic microchip with dried reagents on board. More specifically, all sulfide species (H_2_S, H^S−^ and S^2−^) in water samples are detected by the fluorescence signal emitted upon the reaction with *N*,*N*-dimethyl-phenylenediamine sulfate in the presence of Fe^3+^, leading to the formation of the fluorescent methylene blue (MB) species. It has been proven that the system herein proposed is able to measure sulfide concentration in a linear range from 0–10 mg L^−1^ with a sensitivity value of about 6.7 µA mg^−1^ L and a detection limit of 0.5 mg L^−1^. A comparison with conventional UV-Vis detection method has been also carried out. Data show a very good linear correlation (R^2^ = 0.98093), proving the effectiveness of the method. Results pave the way toward the development of portable and low-cost device systems for water-soluble sulfide pollutants.

## 1. Introduction

Nowadays, the quality of both surface water and groundwater is a crucial task for all countries. In the European Union (EU), the quality of water is monitored by permanent stations in accordance with the water framework directive 2000/60/EC. The EU commitment is well proven by various funded projects focusing on several research aspects related to water pollution control and monitoring, water treatment and management of water resources. 

The development of new nanomaterials and the continuous improvement in the nanotechnologies’ integration has allowed the fast growth of innovative chemical sensors for environmental applications including microbiological surveillance and water monitoring and treatment [1,2,3]. In this context, smart sensors based on multiparametric probes have been extensively developed for water pollutants, including inorganic charged species (nitrate, ammonium, sulfide, nitrite, etc.), heavy metals (Pb, Cd, Cr, Hg, etc.), organic pollution (dioxin, polycyclic aromatic hydrocarbon) and biological pollution (fecal coliform, fecal *Streptococcus*, etc.) [4]. 

In this scenario, there is a growing need to monitor the presence of the water-soluble pollutants of sulfide anion species S^2−^ and HS^−^ that can cause acute and chronic toxicity including neurological effects and at high concentrations, even death [5].

Both of these species are typically produced by human and animal wastes or by bacterial activities. A high concentration of sulfide species is also found in groundwater, especially in the presence of sulfidic hot-springs, typically located in “unconventional environments”, such as inside caves [6]. In this kind of environment, the sample collection and the direct monitoring of the quality of the water are hard and laborious to perform. Moreover, for sulfide species, the fast biotic oxidation requires sample stabilization, typically performed with zinc-acetate form the white and stable zinc sulfide. In this context, various analytical strategies have been developed [7]. The current standard methods for the quantification of sulfide amount are colorimetric or ion-chromatography methods executed in centralized laboratories requiring large equipment and laborious protocols. The development of sensor systems for fast and effective monitoring of sulfide anions in water samples (i.e., 0.5 mg/L for waste water) can provide crucial improvement for point-of-use analysis. In this context, Chen et al. developed a rapid colorimetric method for sulfide detection based on the redox reaction with the formation of gold and gold sulfide nanoparticles [8]. An extensive review on this topic has been written by Pandey et al. [9]. 

Among the proposed literature strategies, those using optical transduction methods are very appealing since they offer high sensitivity (up to nM) and reproducibility, fast response and low cost. Recently, Xiong et al. proposed a miniaturized fiber-optic colorimetric sensor for nitrite determination reaching a detection limit of about 7 μg L^−1^ [10].

The silicon photomultiplier device (SiPM) is a miniaturized photodetector in which every pixel is a single photon avalanche diode (SPAD) operating in Geiger mode [11,12]. The voltage applied generates a strong electric field that allows the single electron-hole pair to trigger the avalanche multiplication of the carriers [13]. The SiPM sensor was recently proposed for DNA detection through fluorophore labelled-DNA strands in microarray technologies [14,15].

Herein, we described a new strategy for fast and sensitive optical detection of sulfide species using a SiPM device coupled with appropriated analytical chemistry in a plastic microchip with reagents on board. More specifically, the analytical method herein employed is based on fluorescence detection obtained upon reaction of sulfide species (H_2_S, H^S−^ and S^2−^) with *N*,*N*-dimethyl-phenylenediamine sulfate in the presence of Fe^3+^, leading to the formation of the fluorescent methylene blue (MB) species [16]. The sensitivity study using analytical samples is presented and discussed. The system has been also tested with real water samples collected in a sulfidic spring, and a comparison with the conventional UV-Vis detection method is finally discussed.

## 2. Materials and Methods

### 2.1. Chemicals

All the reagents used in the reported experiments were purchased from Signal Aldrich (Merck KGaA, Darmstadt, Germany) and used as received. The *N*,*N*-dimethyl-phenylenediamine sulfate solution was prepared dissolving 0.2 g of material in 100 mL of deionized water containing 10 mL of sulfuric acid. The FeCl_3_ solution was prepared dissolving 0.8 g of material in 50 mL of deionized water containing 2 mL of sulfuric acid.

### 2.2. Silicon Photomultiplier System Measurement Setup

**Figure 1:**
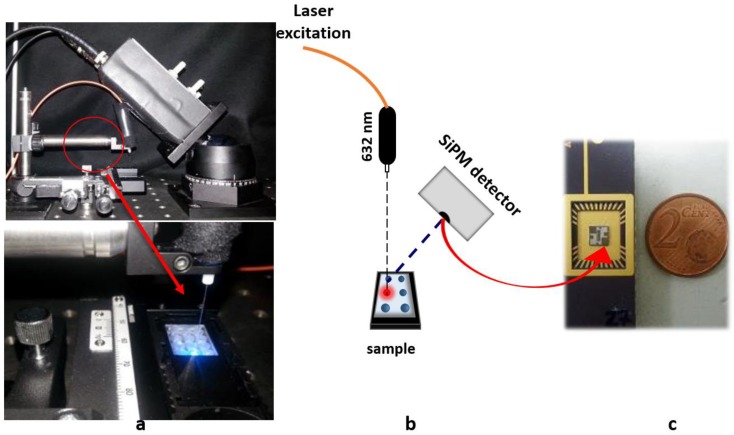
Optical experimental setup: (**a**) detail of the optical system; (**b**) scheme of the geometric setup; and (**c**) the silicon photomultiplier (SiPM) sensor.

The system used for this study is composed of: (a) a SiPM detector, (b) a laser excitation light and (c) a plastic chip (see Figure 1).

(a) The SiPM sensor employed in this work was developed by STMicroelectronics [11]. It is formed of 25 pixels, each one electrically and optically insulated from the others by optical trenches fabricated all around each pixel and filled with silicon oxide. This improvement reduces the device intrinsic noise. For the measurements, it has been mounted in a 32-pin open package (Figure 1c), through which it is possible to bias it and collect the output signal. A Keithley 2636 source meter (Tektronix, Germany) unit was used to bias the SiPM and collect the signal. The signal was recorder by a PC using Labview^®^ (National Instruments, Milan, Italy) and home-made software. The off-line analysis was performed using a home-made MATLAB™ (MathWorks, Natick, MA, USA) routine.

(b) The excitation light at 632.8 nm is provided by a He-Ne fiber-coupled laser. A bandpass filter, centered at 690 nm, is interposed between the sample and the SiPM sensor in order to reduce the optical noise due to the laser radiation reflected from the surface. The measurement system is automated by a customized software.

(c) The plastic chip (17 mm × 13 mm) was made of polycarbonate material. It contains six round-shaped micro-chambers (3 mm in diameter) of 20 μL each. 

For the measurements, the SiPM detector is placed at 45° with respect to both the laser and the plastic chip (see Figure 1).

### 2.3. Sulfide Amount Measurements

For the analytical study, standard solutions containing sulfide amounts from 0–100 mg L^−1^ were prepared dissolving sodium sulfide in deionized water. Twenty microliters of each solution were loaded into the chip microchambers preloaded with *N*,*N*-dimethyl-phenylenediamine sulfate and FeCl_3_ reagents. Upon MB formation, the fluorescence signal was recorded by the experimental setup described in Section 2.2.

The real sulfidic-water samples were collected in a sulfidic spring located in the final gallery of Monte Conca cave. Seven samples from different cave sites containing diverse sulfide concentrations were collected. Each sample of a volume of 20 mL was divided in two aliquots. The first aliquot (10 mL) was stabilized with 1.5 mL of zinc acetate (9.0 g in 30 mL) and stored at room temperature for the sulfide amount measurement by the conventional UV-Vis spectrophotometry method in the laboratory. The second aliquot was dispensed on the miniaturized plastic chips containing the detection reagents on board (*N*,*N*-dimethyl-phenylenediamine sulfate and FeCl_3_). After the reaction, the solution fluorescence at 690 nm was read by the SiPM system according to the procedure described above for the calibration curve.

Each measurement was repeated 5 times. 

The spectrophotometric measurements in the visible range were performed using an Cary 50 spectrophotometer (Varian, Melbourne, Australia).

## 3. Results and Discussion

**Figure 2:**
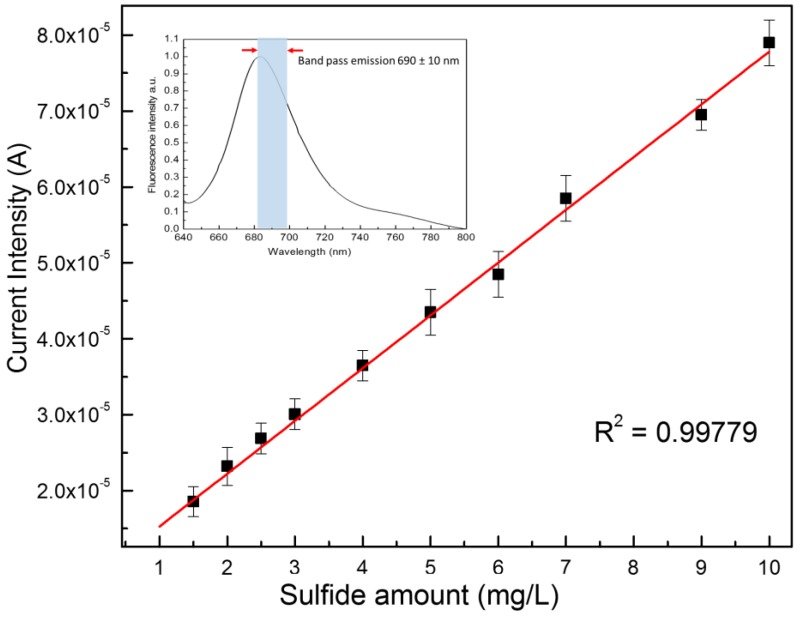
Calibration curve of the SiPM current as a function of the sulfide amount in solution. In the inset, the fluorescence spectra of MB and the filtered emission band at 690 ± 10 nm.

Figure 1 reports the details of the optical system used for this study. The SiPM detector (Figure 1c) is placed at 45° with respect to both the excitation light provided by a He-Ne laser and the plastic microchip containing six microchambers (20 μL each) preloaded with dried reagents and placed at 90° with respect to the laser light. (Figure 1a,b). The sulfide detection in water samples was obtained by measuring the fluorescence emission of MB at 690 nm formed upon the reaction of sulfide with *N*,*N*-dimethyl-phenylenediamine sulfate (NPS) and FeCl_3_ [16]. The inset of Figure 2 reports the emission spectra of the MB molecule, highlighting the emission band at 690 ± 10 nm used for the present study.

The configuration reported above allows the SiPM device to detect the fluorescent signal emitted by the MB molecule.

The first part of the study was focused on the testing of the analytical performances of the system. For this purpose, analytical water samples containing different sulfide concentrations from 0–100 mg L^−1^ were analyzed by the above-described SiPM-based system. The fluorescence values were read as current intensity. 

The collected data showed a linear behavior (sulfide amount versus SiPM current) in the region between 0 and 10 mg/L (see below), while for samples exhibiting sulfide amounts greater than 10 mg/L, a quenching of the fluorescence signal was found (data not shown). 

Figure 2 reports the plot of SiPM current intensities versus sulfide concentrations for the observed linear part occurring between 0 and 10 mg/L of sulfide. Data were interpolated by a linear fit giving the equation Y = 8.3 × 10^−6^ + 6.7 × 10^−6^ X (R^2^ 0.99779). This leads to a sensitivity value of about 6.7 µA mg^−1^ L. The limit of detection (LoD) has been calculated by using a current value corresponding to three-times the background signal (current measured without sulfide analyte). A LoD value of about 0.5 mg L^−1^ (14 μM) was found.

It is noteworthy that the working linear range of the present detection system is better than those obtained by other reported techniques based on the same reaction such as spectrophotometry (LoD: 0.05–2 mg/L [17]), fluorimetry (LoD: 0.75–1.5 mg/L [18]), amperometry (LoD: 0.1–4.8 mg/L [19], electrochemistry (LoD: 0.9 mg/L [20]) and liquid chromatography (LoD: 6.7 × 10^−5^ − 1.5 × 10^−4^ mg/L [21]). Therefore, compared to these techniques, the method herein proposed shows detection performances that are more versatile for applicative use.

Even if the LoD value of our method (14 μM) is lower with respect to other approaches reported in the literature (0.28 μM in [8] and 60 nM in [22]), it is fully in line with the legal requirements for the control of sulfide species in water (0.5 mg/L) [23]. 

Additionally, it is remarkable that, compared to these approaches, the system herein described had the advantage of being used in a portable system for point-of-use analysis.

In order to evaluate the effectiveness of our system for applicative use, real water samples were used. To this aim, seven sulfidic water samples were collected from a sulfidic spring and analyzed. Aliquots (20 µL) of fresh collected water samples were loaded on the microwells of the plastic chip pre-loaded with NPS and FeCl_3_ dried reagents. SiPM current responses were recorded, and the sulfide concentrations were calculated by the linear regression equation reported above. Data are shown in Table 1 (Columns 1–3) and range between 6.4 mg/L and 14.5 mg/L. 

To verify the reliability of the method herein proposed, the same real water samples were also measured by recording the absorbance of methylene blue at 666 nm using a conventional UV-Vis spectrophotometer (Cary 50, Varian, Australia). The sulfide amount was then calculated assuming an absorption extinction coefficient of 70,000 L mol^−1^ cm^−1^ for MB. The obtained data are reported in Table 1 (Column 4) and range between 6.2 mg/L and 16.0 mg/L.

**Figure 3:**
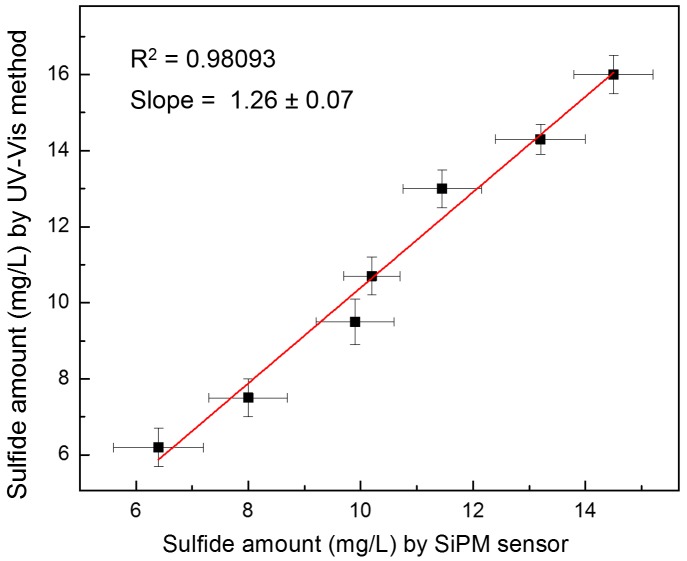
Correlation between the sulfide amount measured by the silicon photomultiplier (SiPM) sensor and the standard UV-Vis method.

By comparing the results reported in Columns 3 and 4 of Table 1, it is possible to note that the data are quite comparable. Figure 3 shows the mathematical correlation between the two analytical methods (correlation curve). A good linear trend is found with a correlation coefficient R^2^ = 0.98093 (slope 1.26 ± 0.07). This indicates that the proposed detection method is very reliable from the analytical point of view and, due to its miniaturized components (SiPM detector, plastic chip), can be certainly considered a very promising prototype for the development of a portable easy-to-use device for fast and effective monitoring of sulfide anions in water samples in point-of-use analysis.

## 4. Conclusions

Sulfide anion species S^2−^ and HS detection is particular relevant in water-soluble pollutants’ monitoring, since they can cause acute and chronic toxicity including neurological effects and, at high concentrations, even death. 

In this study, we have presented a new strategy for fast and sensitive optical detection of sulfide species using an integrated silicon photomultiplier (SiPM) device (developed by ST) coupled with the appropriate analytical strategy applied in a plastic microchip with dried reagents on board. The sulfide species (H_2_S, H^S−^ and S^2−^) present in water samples are detected by the fluorescence signal emitted from MB species formed upon the reaction of sulfide with NPS in the presence of Fe^3+^.

The results herein presented proved that the system is able to measure the sulfide concentration in a linear range from 0–10 mg L^−1^ with a sensitivity value of about 6.7 µA mg^−1^ L and a detection limit of 0.5 mg L^−1^. The comparison with the conventional UV-Vis detection method has shown a very good linear correlation (R^2^ = 0.98093), proving the effectiveness of the method. 

The results pave the way toward the development of portable and low-cost device systems for water-soluble sulfide pollutants. 

## Figures and Tables

**Table 1:** Sulfide concentration in sulfidic water samples measured using the approach proposed in this paper (SiPM) and the standard UV-Vis spectrophotometric method.

Sample	SiPM Current Intensity (µA)	Sulfide mg/L by SiPM	Sulfide mg/L by UV-Vis
1	52.5 ± 2.2	6.4 ± 0.8	6.2 ± 0.5
2	62.9 ± 1.9	8.0 ± 0.7	7.5 ± 0.5
3	77.5 ± 2.0	9.9 ± 0.7	9.5 ± 0.6
4	79.3 ± 2.3	10.2 ± 0.5	10.7 ± 0.5
5 *	87.9 ± 2.1	11.4 ± 0.7	13.0 ± 0.5
6 *	100.0 ± 2.5	13.2 ± 0.8	14.3 ± 0.4
7 *	109.0 ± 2.4	14.5 ± 0.7	16.0 ± 0.5

* Samples 5, 6 and 7 were diluted 1:1 due to the high sulfide concentration. The results reported take into account the dilution.
